# The Impact of COVID-19 on Physical Activity Among Adults in Saudi Arabia: A Cross-Sectional Study

**DOI:** 10.7759/cureus.26586

**Published:** 2022-07-05

**Authors:** Ahmed M Hamed, Haroon A Javaid, Safwan Abbasi, Ahsan Amanullah, Majed Ramadan, Ismail M Shakir, Noara AlHusseini

**Affiliations:** 1 College of Medicine, Alfaisal University, Riyadh, SAU; 2 Research, King Abdullah International Medical Research Center, Riyadh, SAU; 3 Medicine, Alfaisal University College of Medicine, Riyadh, SAU

**Keywords:** cross-sectional study, kingdom of saudi arabia (ksa), saudi arabia, adults, covid-19, physical activity

## Abstract

Background

Physical inactivity has been identified as a major factor in developing and progressing chronic non-communicable diseases such as obesity. The Kingdom of Saudi Arabia ranks high worldwide in rates of obesity. During the coronavirus disease 2019 (COVID-19) pandemic, public health measures have been enforced. These included social distancing, masking, reduction of workplace daily hours, prevention of social gatherings, and home quarantine measures. These ultimately restricted the ability to perform regular physical health activities. The aim of this study is to understand the impact of COVID-19 on physical activity among adults in the Kingdom of Saudi Arabia.

Methodology

A cross-sectional study was conducted among the Saudi population. An online survey was sent through social media to gather data regarding individual physical activity before and after the start of the COVID-19 restrictions. The data were collected from March 20, 2021, until May 20, 2021, and analyzed using chi-square and paired t-test using the SAS software version 9.4.

Results

In total, 433 participants completed the survey. There were 183 (42.3%) males, and the majority of the participants were Saudi nationals (284, 65.6%). Most of the participants (181, 41.8%) were in the age group 25-35 years and 253 (58%) had bachelor’s degrees. Although the results did not show a statistically significant difference between pre- and post-COVID-19 respondents in terms of physical activity, married participants, participants from the eastern province, and participants who did not exercise regularly were all significantly impacted by lack of exercise compared to their counterparts (p < 0.05).

Conclusions

Taking measures to prevent the spread of COVID-19 is essential. Nonetheless, recommendations should be sought for physical activity during lockdowns, and large-scale research should be conducted to better understand what causes the exaggeration of sedentary lifestyles during lockdowns and how to prevent them. Further studies need to be conducted, and national guidelines should be made available in case of a future lockdown.

## Introduction

Physical activity, an essential element for maintaining health, is any bodily movement ‎produced by skeletal muscles that require energy expenditure [1]. Regular exercise should be a pivotal component of the lifestyles of all individuals, regardless of their age and ‎background. On one hand, regularly participating in physical activity has a preventive ‎effect against many diseases. On the other hand, physical inactivity or a sedentary lifestyle ‎is considered to be a risk factor for many of the world’s major non-communicable diseases, such ‎as obesity, cardiovascular disease, type 2 diabetes, and breast and colon cancer [2]. The ‎World Health Organization (WHO) labels physical inactivity as the fourth leading risk ‎factor for global mortality and attributes nearly three million deaths per year to it [3]. A ‎physically inactive lifestyle constitutes a risk to one’s health, which holds possible ill ‎consequences for individuals, families, and communities. According to the North American ‎Nursing Diagnosis Association, one can recognize or diagnose a physically inactive lifestyle ‎through the following three determining factors: choice of a daily routine lacking physical exercise, ‎demonstration of physical deconditioning, and verbalizing preference for activities low in ‎physical activity [4].

A 2011 study examined the prevalence of physical inactivity in 76 countries (80% of the ‎world’s population) and concluded that roughly one-fifth of the population was inactive [5]. ‎While this is a global predicament, Saudi Arabia is not far from reporting alarming ‎statistics. Numerous studies have highlighted the trends in physical activity in Saudi ‎Arabia. The prevalence of physical inactivity within Saudi Arabia ranges between 26% and ‎‎85% among Saudi males and between 43% and 91% among Saudi females [6]. It is also ‎noted that physical activity levels decrease with increasing age in Saudi adults, and this trend ‎is common to both genders [7].‎‎ Apart from gender and age, other factors that have been ‎implicated in influencing activity levels include geographical location and income. The ‎Kingdom of Saudi Arabia suffers from extreme weather status, a lack of parks, and low air ‎quality (dust) that may be a barrier to engaging in physical activity [6]. The central region ‎of Riyadh, Saudi Arabia, lacks physical activity facilities [8], crucial community ‎and neighborhood parks, and organized physical activity programs [9]. In combination ‎with extreme weather, promoting physical activity in the local population is challenging ‎[10].

Physical inactivity leads to the ill outcome of obesity. ‎The causes of obesity have been simplified, by the WHO, as an energy ‎imbalance between calories consumed and calories expended [11]. During environmental ‎and societal changes, a lack of supportive policies results in adverse dietary and physical ‎activity patterns, leading to obesity and other preventable diseases. The Kingdom of Saudi ‎Arabia ranks high among countries that suffer from obesity [12]. In 2020, a world ‎population review revealed that almost 35% of the Saudi population suffers from obesity. In ‎a recent study, obesity in adult females working in offices was reported to be 58% [13], while ‎another reported that 67% of adult males suffer from obesity in the Kingdom of Saudi ‎Arabia [14]. Within the Kingdom, in both public and private sectors, public awareness has been ‎initiated to tackle the burden that obesity might pose on the population’s health. However, ‎minimal implementation was observed in all published articles in various cities in the ‎Kingdom as no prominent policies were devised to solve this issue. The government, its ministries, and the community face multiple barriers while solving the problem.

The WHO announced a global pandemic caused by coronavirus disease 2019 (COVID-19) in ‎March 2020. Owing to the home confinement imposed by numerous countries worldwide ‎and the understandable fear of the pandemic, physical activity levels plummeted. For ‎instance, a study by the WHO in a sample of 10 countries found a mean decrease of 5.5% ‎in physical activity levels (using daily step count as a proxy for this measure) within ‎‎10 days of the declaration of COVID-19 as a pandemic. This number increased to 27.3% within ‎‎30 days [15]. Daily, the time spent sitting was reported to have increased by two ‎hours [16]. This also correlated with higher depression or anxiety scores [17]. Moreover, a marked ‎increase in body mass index (BMI) was reported [18]. Furthermore, more individuals reported not experiencing ‎good sleep during the quarantine compared to before [16]‎.

In the Kingdom of Saudi Arabia, the first case of COVID-19 was confirmed on March ‎‎2, 2020. On March 8, all educational institutions were suspended, and E-learning programs ‎were initiated [19]. Attendance was suspended in all government offices. Health clubs and ‎private gymnasiums closed down. Restaurants and cafes were only allowed to offer delivery ‎services. Social events such as weddings and funerals were also prohibited. Curfews were ‎implemented nationwide and in various cities several times during March and ‎April. International flights were halted. Domestic travel via flights, buses, and trains was ‎restricted. As the number of cases gradually decreased, on June 21, all restrictions ‎were lifted through a phased program except for social distancing and wearing ‎masks in public spaces [20].

Compared to the rest of the world, similar effects on lifestyle and health behavior have ‎been studied and reported among the Saudi population during the lockdown period in a ‎few studies [17,21-25]. Our goal was to investigate the impact of the global pandemic and ‎public health restrictions on adults’ physical activities among various demographics in ‎Saudi Arabia. Besides filling potential gaps in the literature, this study aimed to identify ‎potential solutions to the public health burden of physical inactivity among adult residents ‎of Saudi Arabia during the COVID-19 pandemic and outline any trends to help recommend ‎guidelines for future lockdowns. This coincided with the government’s Quality of Life aim ‎within the Kingdom’s Vision 2030.‎

## Materials and methods

Study design

This was a cross-sectional study. An online survey was distributed via social media channels among the Saudi population. Convenience sampling and snowball sampling techniques were used for data collection. Snowball sampling involves finding an individual, collecting information from that ‎individual, and asking him/her to refer others to the study. It may occur in multiple stages ‎and help recruit hard-to-reach individuals [26]. ‎ After filling out the survey, participants were asked to refer the study to others. The data were collected using a questionnaire distributed through a survey administration tool, Google Forms. A sharable link was built to ease the method of distribution among the desired population. The survey distribution, from March 20, 2021, until May 20, 2021, was conducted via email and social media networks such as WhatsApp, Telegram, Facebook, Twitter, and LinkedIn.

Sample size

Yamane’s formula was used to calculate the sample size of the target population [27]:

$$n=N/(1+N(e)^{2}) =26456921/(1+26456921(0.05^{2}) =26456921/66143.3025 =399.993952 \approx 400$$

To achieve a confidence level of 95% with a margin of error of 5%, the formula calculated the required sample size as 400. Statistical significance was determined at a p-value of <0.05. According to the most recent information by the General Authority for Statistics in Saudi Arabia, the population of people above the age of 18 was found 26,456,921 [28]. Both genders were expected to participate equally.

Questionnaire development

Questions were adapted from the Godin-Shephard Leisure-Time Physical Activity/Exercise Questionnaire (GSLTPAQ) [29] and a similar study conducted on Canadians [30]. The questionnaire was in a survey format and comprised the following two sections: (1) Demographics: age, gender, the highest level of education, marital status, nationality, monthly income, employment before and during COVID-19, region of residence, and history of any medical conditions. (2) Physical activity:** **The GSLTPAQ was developed by Godin in Canada in 1985 to measure and assess the degree of physical activity adults partake in during their leisure time. GSLTPAQ is an internationally used and accredited tool used to compute a score that corresponds and correlates to a certain level of physical activity/exercise [29]. Physical activity behaviors before and during COVID-19 were determined. The following equation from GSLTPAQ was adapted: Weekly leisure-time activity score = (9 × Strenuous) + (5 × Moderate) + (3 × Mild).

The result of this equation was interpreted as described in Table 1. Active participants were recognized to have achieved substantial benefits from their respective physical activity routines. Moderately active participants were recognized to have achieved some benefits from their respective physical activity routines. Insufficiently active individuals had less substantial or low benefits achieved from their habits.

**Table 1:** Scoring system for the Godin-Shephard Leisure-Time Physical Activity/Exercise Questionnaire.

Godin Scale score	Interpretation
24 units or more	Active
14–23 units	Moderately active
Less than 14 units	Insufficiently active/Sedentary

Pilot study

A pilot survey was conducted on 10 participants. Pre-testing questionnaires contributed to the validity and reliability of questionnaire survey evidence. To investigate how participants responded to the questionnaire, a cognitive interviewing method was used. Experts in the field of public health were involved to achieve content validity.

Ethical consideration

Permission to collect data was obtained from the Institutional Review Board (IRB) at Alfaisal University. The identities and personal information of respondents were kept anonymous. The data were saved in a secure device that was only accessible by the research team. An introduction paragraph at the start of the questionnaire assured the participants about data confidentiality, and the survey did not ask for respondent names.

Data analysis

Descriptive statistics were used to examine the differences in baseline demographic characteristics among participants. For univariate analyses, chi-square tests, Fisher exact tests, and paired t-tests were used where appropriate to examine the participants’ characteristics. To examine the mean score of the GSLTPAQ difference before and during the pandemic, or the impact of the COVID-19 pandemic on physical activity, multivariate analysis using the generalized linear model (GLM) adjusted for potential demographic confounders was employed. Homogeneity of variance and normality of residuals assumptions were checked and no assumption violations were observed. A p-value of <0.05 was considered statistically significant for the multivariate analyses. All statistics and data analyses were performed using SAS software version 9.4 (SAS Institute Inc., Cary, NC, USA) [31].

## Results

Demographics

The study included 433 participants, of which 42.3% were males. The participants’ age ranged from 18-65, with the majority of the participants in the 25-35-year age group (41.8%), followed by the 18-24-year age group (37.4%). Regarding the educational level, most participants had a bachelor’s degree (58.4%). Single people made up the majority of our study population (62.4%). The predominant BMI group was 18.5-24.9 kg/m^2^ (45.7%), classified as normal weight. This was followed by 30% of the participants in the overweight category (30%), with a BMI ranging from 25-29.9 kg/m^2^. Most of the participants included in the study were Saudi (65.6%) and resided in Riyadh/central region (53.6%). Upon assessment of their income status, 36.6% of the participants had no income, while 18.2% had an income of less than 10,000 Saudi Riyals. Table 2 shows the detailed sociodemographic characteristics of the participants.

**Table 2:** Sociodemographic characteristics of the participants (n = 433‎).

Variables	Frequency (n)	Percentage (%)
Gender		
Male	183	42.3
Female	250	57.7
Age group		
18-24 years	162	37.4
25-35 years	181	41.8
36-45 years	57	13.2
46-55 years	22	5.1
56-65 years	11	2.5
Education level		
Bachelor's degree	253	58.4
Graduate degree	91	21.0
High school, diploma, or less	89	20.6
Marital status		
Single	270	62.4
Married	148	34.2
Divorced	10	2.3
Widowed	5	1.2
Body mass index		
<18.5	25	5.8
18.5-24.9	198	45.7
25-29.9	130	30.0
>30	80	18.5
Nationality		
Saudi	284	65.6
Non-Saudi	149	34.4
Place of living		
Northern region	12	2.7
Southern region	57	13.2
Eastern region	35	8.1
Western region	78	18
Central region	251	58
Monthly income (SR)		
9,999 Saudi Riyal or less	79	18.2
10,000-14,999 Saudi Riyal	67	15.4
15,000-19,999 Saudi Riyal	28	6.5
20,000 or more Saudi Riyal	45	10.4
Do not have an income	159	36.6
Prefer not to answer	55	12.7

**Figure 1:**
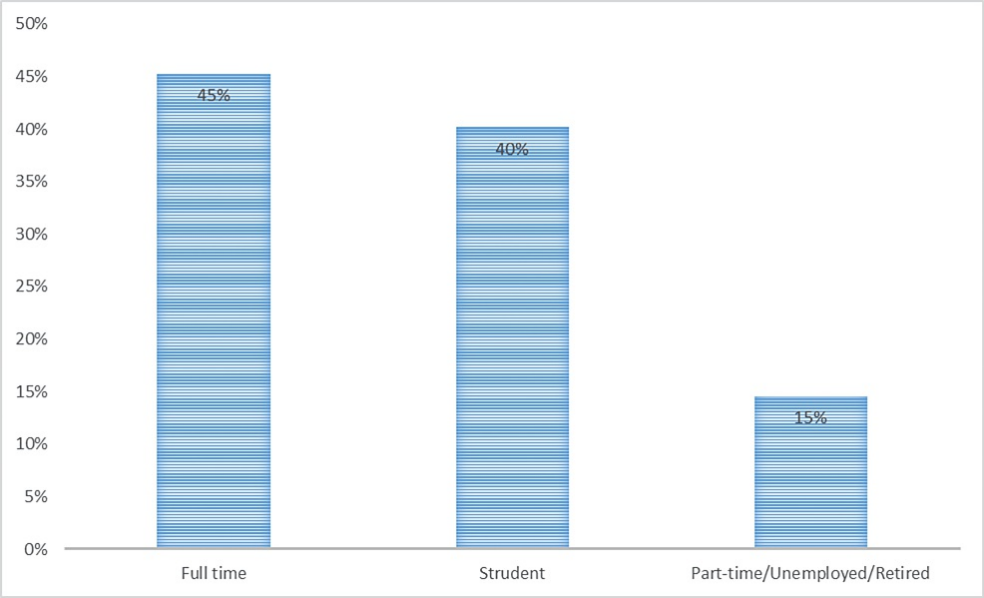
Bar chart showing the employment status before COVID‎-19. COVID-19: coronavirus disease 2019

**Figure 2:**
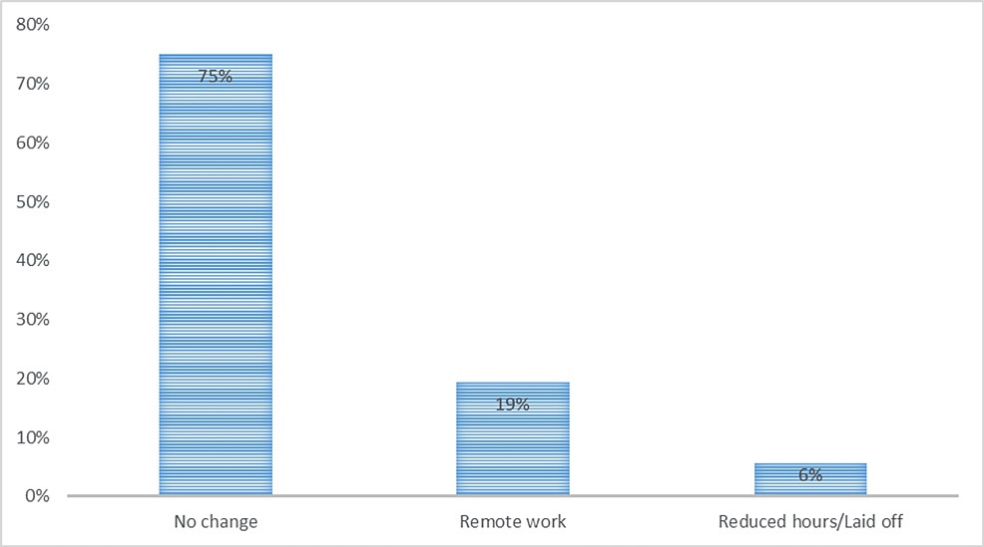
Bar chart showing the employment status during COVID-19. COVID-19: coronavirus disease 2019

With regards to the employment status, before the COVID-19 pandemic, 196 (45.3%) participants were full-time employed, while 174 (40.2%) were students. The remaining participants (57, 13.2%) were part-time employees, and six (1.4%) were retired. During COVID-19, 325 (75.1%) participants had no employment changes. However, 84 (19.4%) participants started working remotely, 16 (3.7%) had reduced working hours, and eight (1.8%) were laid off. Figure 1 and Figure 2 depict the employment status before COVID-19 and the change in employment status due to the COVID-19 pandemic.

Physical activity: the Godin-Shepard Leisure-Time Score

In total, 433 participants were evaluated, of whom 79 had health issues. Of those, 24 (5.5%) had undergone past surgeries, seven (1.6%) had hypertension, and six had hypertension and diabetes or diabetes alone (1.4%). Three (0.7%) participants had injuries, and 34 (7.9%) had comorbidities that were not specified.

**Figure 3:**
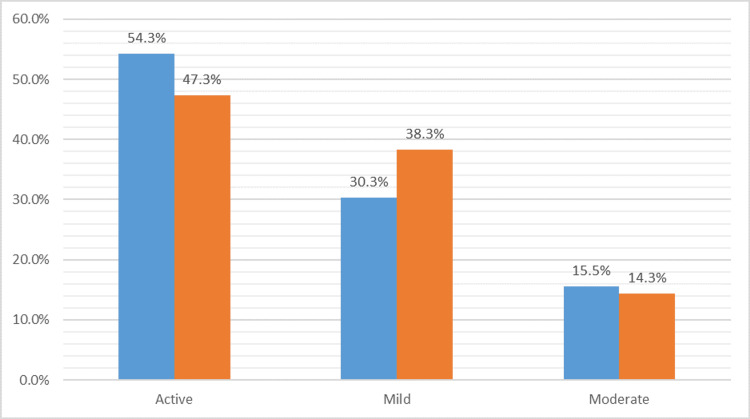
Bar chart illustrating the percentage of individuals in each group of activity before COVID-19 and during ‎COVID-19‎. COVID-19: coronavirus disease 2019

The weekly leisure time activity score was calculated using the GSLTPAQ for participants before COVID-19 and during COVID-19 for each participant. The number of active participants scoring 24 or higher before COVID 19 was 235 (54.3%), which was reduced to 205 (47.3%) during the COVID-19 pandemic. Furthermore, the number of moderately active individuals scoring between and including 14 and 23 before COVID-19 was 67 (15.5%), which was reduced to 62 (14.3%). Likewise, insignificantly active participants scoring 13 or fewer before COVID-19 was 131 (30.2%), which increased to 166 (38.3%). Figure 3 illustrates the percentage of weekly leisure time activity scores before and during the COVID-19 pandemic.

**Figure 4:**
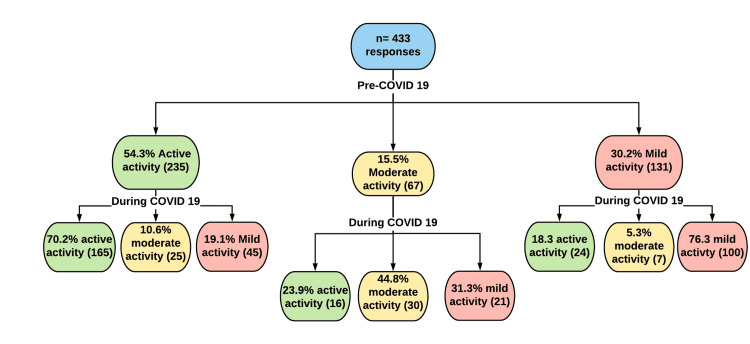
Flowchart showing the impact of COVID-19 on each group of leisure time activity. COVID-19: coronavirus disease 2019

During the pre-COVID-19 period, 235 individuals in the active group participated in the study, with 165 (70.2%) remaining active during the COVID-19 pandemic. However, 25 (11.1%) participants went from active to moderately active, while 45 (20%) went all the way to mildly active during the COVID-19 pandemic. In addition, among the 67 (15.5%) participants in the moderately active group, 30 (44.8%) remained at the same activity level during the COVID-19 pandemic. However, 25 (37.3%) participants increased their level of activity to become active, while 21 (31.3%) reduced their level of training to mildly active during the COVID-19 pandemic. Nevertheless, among the 131 individuals in the insufficiently/mildly active group, 100 (76.3%) remained insufficiently active during the COVID-19 pandemic. Despite this, 24 (18.3%) participants went from insufficiently active to active, while seven (5.3%) went all the way to mildly active during the COVID-19 pandemic. Figure 4 displays the changes that occurred in each group during COVID-19.

**Figure 5:**
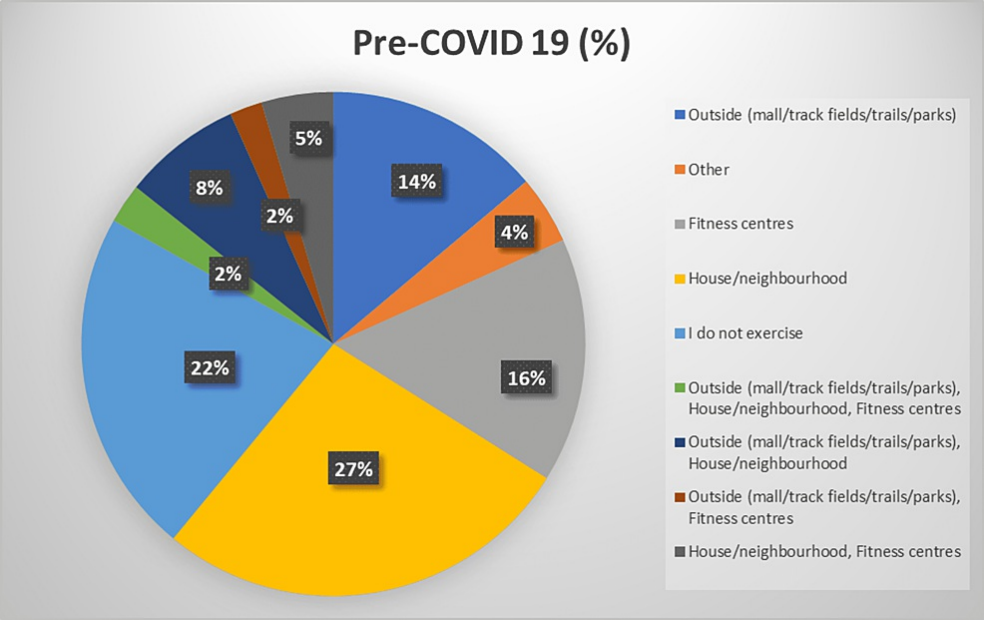
Pie chart depicting locations of exercise before COVID-19. COVID-19: coronavirus disease 2019

**Figure 6:**
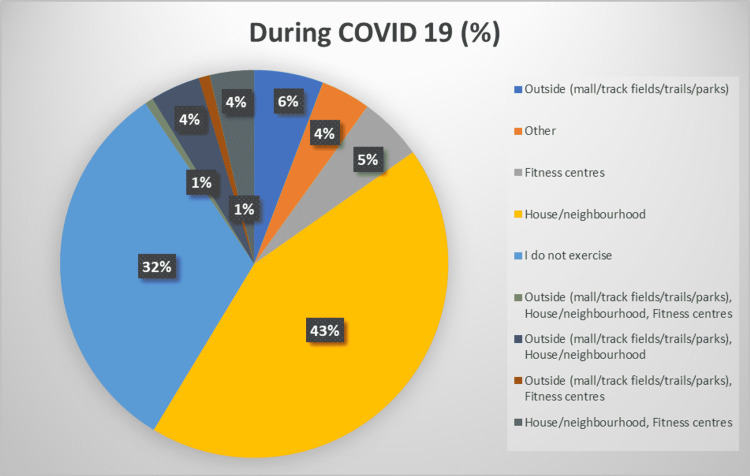
Pie chart depicting locations of exercise during COVID-19. COVID-19: coronavirus disease 2019

During the pre-COVID-19 period, most of the participants (112, 25.9%) exercised at home or in their neighborhood, while 96 (22.2%) did not exercise. However, 65 (15%) participants exercised at a fitness center, while 57 (13.2%) and 19 (4.4%) exercised outside and in other locations, respectively. On the other hand, during COVID-19, the trend remained the same where the largest number of individuals would exercise at home or in their neighborhoods, followed by “no exercise” and “other” locations. It is worth noting that, despite the locations having not changed in ranking, all locations witnessed a reduction in the number of people visiting them for exercise except homes and neighborhoods. Figure 5 and Figure 6 better depict the locations where people would visit to exercise before and during the COVID-19 pandemic.

Comparative analysis

In multivariate analysis adjusted for potential confounders, there was no statistically significant difference in the Godin-Shepard Leisure-Time scores between before and during the COVID-19 pandemic. The only significant predictor of the overall physical activity score was marital status. There was a statistically significant difference in the overall mean scores of physical activity between married and unmarried respondents. Married individuals reported a lower physical activity score compared to unmarried respondents (beta coefficient = -0.13; p = 0.0001). Respondents who reported not exercising regularly and those who preferred not to say both reported lower overall physical activity scores when compared to respondents who exercised regularly (beta coefficient = -0.59; p < 0.0001; beta coefficient = -0.41; p = 0.02, respectively). Respondents from the eastern region reported lower physical activity scores compared to the western region (beta coefficient = -0.37; p = 0.004). Table 3 presents these findings in detail.

**Table 3:** Godin-Shephard Leisure-Time Physical Activity score comparison (before and during the ‎COVID-19 pandemic) multivariate analysis. ^1^General linear model. COVID-19: coronavirus disease 2019

	Estimate (β coefficient)^1^	Standard error (SE)	P-value
GSLTPAQ score			
Before COVID-19 pandemic	Reference	Reference	Reference
During COVID-19 pandemic	-0.088	0.059	0.13
Age (year)			
18-24	-0.15	0.21	0.46
25-35	-0.05	0.203	0.78
36-45	0.04	0.21	0.84
46-55	0.2	0.23	0.38
56-65	Reference	Reference	Reference
Gender			
Male	Reference	Reference	Reference
Female	-0.024	0.064	0.69
Marital status			
Married	-0.31	0.08	0.0001
Not married	Reference	Reference	Reference
Nationality			
Saudi	Reference	Reference	Reference
Non-Saudi	0.016	0.07	0.82
Education			
High school, diploma, or less	Reference	Reference	Reference
Bachelor’s degree	-0.058	0.087	0.51
Graduate degree	-0.13	0.11	0.26
Comorbidity			
Yes	Reference	Reference	Reference
No	0.015	0.086	0.85
Body mass index			
Mean	-0.005	0.0058	0.33
Exercise regularly			
Yes	Reference	Reference	Reference
No	-0.59	0.064	<0.0001>
Prefer not to say	-0.41	0.18	0.02
Monthly income			
10,000-14,999 Saudi Riyal	-0.19	0.11	0.09
15,000-19,999 Saudi Riyal	-0.13	0.15	0.4
20,000 or more Saudi Riyal	0.21	0.13	0.11
9,999 Saudi Riyal or less	0.04	0.11	0.65
Do not have an income	0.05	0.1	0.61
Prefer not to answer	Reference	Reference	Reference
Region of residence			
Riyadh/Central	0.03	0.09	0.67
Eastern	-0.37	0.13	0.004
Southern	-0.017		0.11
Western	Reference	Reference	Reference
Northern	0.11	0.2	0.6

## Discussion

The results of this study showed a decrease in the amount of exercise during COVID-19 among the population of Saudi Arabia. However, this was not statistically significant. During the lockdown and due to working from home, a study conducted among 486 young and middle-aged Saudi adults reported that there was ample time for physical activity compared to before the COVID-19 pandemic [22]. However, in this study, the number of active people decreased from 54.3% to 47.3% throughout the quarantine period. Nonetheless, all demographics showed an overall decrease in the active group and an increase in the mildly active group, except in the divorced and widowed demographic which had a slight increase in activity levels and a slight decrease in the mildly active group and the age group 46-55 which showed no change in the active group.

In this study, the possible cause of the witnessed decrease of 51% is that the studied population used outdoor facilities to aid them while exercising and that strict lockdown policies during the pandemic limited their access to many facilities. Although there was an increase in the percentage of people exercising indoors and in neighborhoods, a 10% increase in those who claimed to not indulge in physical activity was also noted. These results are consistent with recent studies by Bawardis et al. and Abdulsalam et al. from the Kingdom of Saudi Arabia, studying 486 young and middle-aged Saudi adults and 472 adults, respectively, and another study by Sañudo et al. from Spain among 139 young adults before and during the COVID-19 quarantine [17,25,32]. Additionally, a study conducted among 5,896 participants from 17 countries in the Middle East and North Africa (MENA) region reported that over one-third of the participants stopped practicing any form of physical activity, and well over half of the studied population spent more time on social media [33]. On the other hand, a study conducted in Canada by Lesser et al., which analyzed the impact of health restrictions during COVID-19 on the population’s physical activity, noted that the most inactive participants reported less physical activity, and the majority of active participants reported more physical activity [30].

An earlier study suggested that males in Saudi Arabia exercise three times more than females [7]; however, in this study, we found that males and females reported similar levels of physical activity before the pandemic at 56.8% and 52.4%, respectively. Nevertheless, the outcomes of this study revealed that the physical activity levels of females were less impacted by COVID-19 measures compared to males. There was a 2.4% reduction in the level of female activity compared to the 13.1% reduction in the active group among males. The witnessed physical activity decrease in the male group was more than five-fold the decrease in females. However, this decrease was not statistically significant in this study. Meanwhile, a study on the impact of COVID-19 on 244 young and middle-aged Saudi adults’ physical activity only in the Western province of Saudi Arabia reported similar but significant outcomes. Among males and females, there was a significant decrease in the physical activity performed, with males slightly higher at 58% decrease and females showed a decrease of 55% [22].

Additionally, in this study, the age ranged between 18 and 65, and all age groups witnessed a decrease in the active group and an increase in the mildly active group, apart from the age group of 46-55 which showed no change in the active group. Though a significant p-value was not associated with the difference in activity level before and during COVID-19. Because the majority of the age groups were impacted negatively, these indicate that age did not play a role in the impact of COVID-19 on physical activity. However, it is noteworthy to mention that most of the participants of this study were between the ages of 18-45 accounting for 92.4% of the total study population.

A study conducted in Saudi Arabia with a sample size of 472 and an age range of 18-59 showed Saudis are more likely to be impacted negatively by the COVID-19 pandemic. Furthermore, in the same study, non-Saudis were more likely to increase their physical activity during quarantine [24]. Conversely, this study’s results showed Saudis were less impacted by the COVID-19 pandemic when compared to non-Saudis. Although no significance was noted, the sum of Saudis in the active group before the COVID-19 pandemic was 52.5% which was reduced to 47.6% (reduced by 4.6%) during the COVID-19 pandemic, and the sum of non-Saudis in the active group was 57.7% which was reduced to 40.9% (reduced by 11.4%). The impact was negative on both the Saudi and non-Saudi groups, yet the non-Saudi group was reduced by more than double than that of the Saudi group.

Our study found significantly lower physical activity scores among married individuals. These findings correlate with a similar study that highlighted barriers to physical activity during quarantine among adults in Saudi Arabia. They found that married individuals had lower odds of engaging in regular physical activities. Interestingly, being married was found to be a factor in choosing walking only as an exercise method in the study. Spousal and family responsibilities were given priority and thus become barriers to regular physical activities [34]. A study conducted prior to COVID-19 found married men to exercise less often as well. Lack of time and facilities were reported as the most important reasons for being physically inactive [35].

These results are in contrast to similar studies conducted in other countries. A Chinese study focusing on home-based exercise among 449 individuals found that married individuals were significantly more inclined to exercise at home during the pandemic. A possible explanation given was that a spouse may be an easy exercise partner [36]. A similar study examining changes in habits during the pandemic in approximately 2,700 adults in the United States found that individuals who were never married had higher odds of decreasing exercise time than married or those living with a partner [37].

Looking further into the results of this study, it was noted that individuals in the eastern region of Saudi Arabia reported lower levels of activity compared to the western region. A cross-sectional study focusing on physical activity at home was conducted in two cities of Mecca and Madina, which are considered part of the western region. The study reported a significant decrease of 57.1% in time spent on the physical activity before and during the pandemic [22]. However, no similar studies have been found regarding the eastern region. A 2015 study attempted to determine the sociodemographic correlations with physical inactivity in the Saudi population. It reported the highest levels of physical inactivity in the northern and central regions and the lowest in the southern region. Mountainous terrain, agricultural predominance, and lack of urbanization in the south were mentioned as possible factors [21]. To the best of our knowledge, this is the first study to find statistically significant differences between these different regions of Saudi Arabia during the pandemic. Possible reasons for these differences include the nature of complete or partial lockdowns in different regions, lack of availability of sports and fitness centers, and increased working from home. Potential future studies should focus on highlighting the differences in physical activity levels between different regions of the country, as well as identifying specific barriers that led to this disparity.

Public health restrictions, such as lockdowns, are likely to influence physical activity behavior on more than one level given the multiple dynamic factors that influence it. Possible causes include lack of access to facilities due to the lockdown, the psychological impact of COVID-19, lack of awareness of the importance of physical activity in circumstances like a lockdown, and spending more time on sedentary activities such as screen time, sleeping, and many more [17,32]. Further research is needed to identify the global and local causes of the negative impact and to help allocate a possible resolution in the event of future lockdowns.

Study limitations

A larger study group with a longer period to collect data may result in more accurate results. Although the time of data collection was during the COVID-19 lockdown in the Kingdom of Saudi Arabia, part of the study design aimed to collect data before the COVID-19 lockdown; hence, recall bias may play a role. In addition, questionnaires distributed online restricted respondents to those with Internet access. Hence, using snowball sampling for those without Internet access and using different methods for recruiting participants may help in a diverse demographic representation. Furthermore, utilizing an international activity scale may not reflect accurately on local participants; hence, more recent and local-scale investigations should be conducted and used to ensure more accurate data. Finally, the study should have addressed more variables that may influence physical activity and should have used a more objective scale to achieve a measurable and reliable result.

## Conclusions

Preventing the spread of COVID-19 was a crucial act that was taken ‎seriously within the Kingdom of Saudi Arabia, yet with every decision, there is a cost. ‎Fortunately, the cost of an increase in a sedentary lifestyle is reversible. Although the ‎outcomes were not statistically significant, this study’s findings have shown a decrease in ‎physical activity among Saudi residents due to the COVID-19 lockdown.

Given the multifactorial nature of engaging in physical activity, different approaches can ‎make a remarkable change in reducing the impact of future lockdowns in the Kingdom of Saudi Arabia. These ‎include benchmarking a nationwide requirement to meet and maintain a healthy lifestyle in ‎terms of physical activity during lockdowns, as well as increasing awareness of the benefits of physical activities ‎via several tools, such as social media, radio, and television. Fitness centers and influencers may be ‎used to promote indoor physical activities. Governmental and non-governmental organizations such ‎as the Ministry of Sports may host virtual games which offer incentives to ‎winners, with objectives to increase physical activity engagement, such as engagement in virtual games or ‎counting the number of steps taken per day. Further and more thorough research on how the ‎COVID-19 lockdown impacted physical activity is recommended, along with further research ‎on the possible incentives for engaging in physical activities. This will allow the use of ‎social, financial, mental, and cultural incentives among relevant groups to reduce the ‎negative impact of such future lockdowns.
